# Micro-computed tomographic analysis of the morphology of maxillary lateral incisors

**DOI:** 10.1007/s00784-024-05727-x

**Published:** 2024-05-23

**Authors:** Thomas Gerhard Wolf, Theodora Rempapi, Sven Schumann, Guglielmo Campus, Gianrico Spagnuolo, Niccolò Giuseppe Armogida, Andrea Lisa Waber

**Affiliations:** 1 Department of Restorative, Preventive and Pediatric Dentistry, School of Dental Medicine, University of Bern, Bern, Switzerland; 2 Department of Periodontology and Operative Dentistry, University Medical Center of the Johannes Gutenberg University Mainz, Mainz, Germany; 3 Institute for Anatomy, Johannes Gutenberg University Mainz, Mainz, Germany; 4 Department of Cariology, Saveetha Dental College and Hospitals, SIMATS, Chennai, India; 5 Department of Neurosciences, Reproductive and Odontostomatological Sciences, University of Naples Federico II, Napoli, Italy

**Keywords:** Analysis, intern morphology, Maxillary lateral incisor, Micro-computed tomography, Root canal configuration

## Abstract

**Objective:**

This study aimed to investigate the morphology of maxillary lateral incisors (MxLI) using micro-computed tomography (micro-CT).

**Materials and methods:**

The root canal configurations (RCC) of maxillary lateral incisors (MxLI) of a mixed Swiss-German population were examined using micro-CT, 3D imaging, and a 4-digit system code indicating the main root canal from coronal to apical thirds and the main foramina number.

**Results:**

The most frequently observed RCC of MxLI were 1-1-1/1 (Vertucci I/Ve I, 80.0%), 1-1-2/2 (Ve V, 7.3%), 1-2-1/1 (Ve III, 6.4%), 2-1-1/1 (Ve II, 1.8%), and 1-1-1/2 (1.8%)(*n* = 110). Three additional RCC were observed less frequently (0.9%). The MxLI showed one physiological foramen in 89.1%, two in 9.1%, and seldom three (1.8%). Most accessory canals were identified in the apical third of a root (20.0%), and no accessory canals in 72.7% of the samples.

**Conclusions:**

Detailed information on the internal morphology of MxLI of a Swiss-German population is given. The most frequently observed RCC of MxLI is 1-1-1/1 (Ve I). However, accessory canals may occur in all apical thirds, and 20% of all teeth investigated showed a challenging RCC for clinical treatment.

**Clinical relevance:**

This study offers clinicians comprehensive data on MxLI morphology, emphasizing the significance of understanding varied RCC and accessory canal presence for improving root canal treatment outcomes. Over 25% of teeth exhibited complex RCC or accessory canals, influencing decisions during root canal treatment.

## Introduction

The success of endodontic non-surgical and surgical treatment depends on the dentist’s accurate three-dimensional knowledge of the internal tooth morphology to access microorganisms and pulp tissue and properly treat the root canal system and physiological foramen area [1, 2]. Due to the morphological differences of each tooth, the requirements and challenges of preparation, disinfection, and obturation of the root canal system depend on the anatomical or morphological characteristics of the individual tooth to be treated [3, 4]. Not only the different tooth types in the human dentition but also maxillary lateral incisors have already been investigated in various studies and possible anatomical variations have been pointed out [1, 5–13]. However, to the best of our knowledge, no study has used micro-computed tomography to investigate the internal morphology or root canal configuration of maxillary lateral incisors (MxLI) yet. In this regard, micro-CT imaging with rendering and software imaging is a tooth structure-preserving, noninvasive, and replicable ex vivo technique for understanding the complexity of the internal morphology of the root canal system [14–16]. Several research groups have introduced classification systems for root canal configurations [1, 17–19]. However, several complex root canal configurations (RCC) cannot be accurately defined using, for example, the classification methods of Weine et al. [17] or Vertucci [1]. The RCC description proposed by Briseño Marroquin et al. [18] divides the root canal pathway into thirds and defines the number of major foramina using a four-digit code system. The present study aimed to examine the morphology and root canal configuration of maxillary lateral incisors of a mixed Swiss-German population using micro-computed tomography.

## Materials and methods

### Tooth selection

Extracted human permanent maxillary lateral incisors (MxLI) were collected from dentists and dental clinics in Germany and Switzerland for reasons not related to this study and stored in a 3% chloramine solution. A sample size calculation of teeth that were extracted in a period of 6 months from 1 June 1 to 31 December 2022, for reasons unrelated to this study that could be used for evaluation according to the above criteria was calculated using a proportion test with a confidence level of 95% and an expected prevalence of 90%. To the sample size of *n* = 97 .5 teeth were added in case of unexpected artifacts that would have led to an impossible analysis or failure [19]. The samples were cleaned of calculus or tissue remnants using manual and ultrasonic scalers. They were then placed in an ultrasonic bath containing 3% hydrogen peroxide solution for one hour and then stored in a 70% ethanol solution. All teeth that were investigated in this study were declared as “excess material”, thus, they could be used for scientific purposes without requiring any additional approval of the corresponding ethics committee (Contract General Terms [AVB], § 14 Organ explantation/further use of body material, Status: 1 April 2017). All the teeth investigated were independently selected by two observers (T.G.W., A.L.W.). A third independent observer could be consulted in case of inconclusive determination (T.R.). The selection criteria for the specimen were complete coronal and root development, no signs of root fracture or resorption, no radicular or coronal caries, and no endodontic treatment. Only MxLI that could be clearly identified as maxillary lateral incisors [20] were considered in the current study or were discarded, otherwise. Despite clearly defined geographical origin, statistical calculation of the sample size, and probable pathologic health status of the teeth, a representativeness of the sample cannot be clearly assigned due to missing information on age and gender, defined exclusion criteria, and missing micro-CT comparison studies. A total of 110 human permanent maxillary lateral incisors (MxLI) were included in this study.

### Morphologic analysis

The MxLI were scanned at an isotropic resolution of 16 μm in a desktop micro-computed tomography unit (µCT 40; SCANCO Medical AG, Brüttisellen, Switzerland) at settings of 70 kV and 114 mA, resulting in 800–1200 slices per tooth. For differentiation of tooth structures, the obtained images were visualized and illustrated using 3D rendering software (VGSTUDIO Max 2.2; Volume Graphics, Heidelberg, Germany) by displaying them with the use of dummy colors. The pulp chamber and the root canal system were colored red, the enamel and crown areas white, and the root and dentin areas with transparent grey. To be able to describe the root canal configuration (RCC), a 4-digit system code was used [18]. The number of root canals is divided from the enamel-cement boundary to the apex into the three parts coronal, middle, and apical and thus described by the first 3 digits of the 4-digit code. The fourth digit, separated by a slash, indicates the number of pertaining physiological foramina. The foramina belonging to the same root canal and having a diameter of at least 0.1 mm were defined as physiological foramina. In addition, the number of observed accessory canals was also recorded. The results are given as absolute and relative values.

## Results

The root canal configuration (RCC) results are shown in Table 1. All examined maxillary lateral incisors (MxLI) in this report were single-rooted. Overall, eight different RCC were investigated. The most common RCC using the Briseño Marroquín et al. classification system [18] in MxLI was 1-1-1/1 (Ve I, 80.0%), followed by 1-1-2/2 (Ve V, 7.3%), 1-2-1/1 (Ve III, 6.4%), 1-1-1/2 (1.8%) and 2-1-1/1 (Ve II, 1.8%). 1-1-2/3 (0.9%), 1-1-3/3 (Ve VIII, 0.9%), and 1-3-1/1(0.9%) occurred less frequently. 15.4% of the samples had other RCC that could not be assigned to a Vertucci [1] or Weine et al. [17] classification. The number (n) and mean (%) of accessory canals and main apical foramina observed in the coronal, middle, and apical third of maxillary lateral incisors are shown in Table 2.

**Table 1:** Root canal configuration of 110 maxillary lateral incisors by means of micro-CT. The RCC are depicted according to the classifications of Weine et al. (We) [17], Vertucci (Ve) [1] and Briseño-Marroquín et al. (Br) [18], with the later classification method describing the root canal configuration from left to right from the coronal, middle and apical thirds of the root. The fourth number is separated with a slash (/) and shows the physiological foramina number [18] (n^total^=110)

Root Canal Configuration			(*n*)	(%)
Br	We	Ve		
1-1-1/1	I	I	88	80.0
1-1-1/2			2	1.8
1-2-1/1		III	7	6.4
2-1-1/1	II	II	2	1.8
1-1-2/3			1	0.9
1-1-2/2		V	8	7.3
1-1-3/3		VIII	1	0.9
1-3-1/1			1	0.9
**Total**			**110**	**100.0**

**Table 2:** Number (n) and mean (%) frequency of accessory canals and main apical foramina observed in the coronal, middle, and apical third of maxillary lateral incisors (n^total^=110)

Accessory canals	(*n*)	(%)
None	80	72.7
Coronal third	1	0.9
Middle third	7	6.4
Apical third	22	20.0
**Main apical foramina**	**n**	**%**
/1	98	89.1
/2	10	9.1
/3	2	1.8

**Fig. 1:**
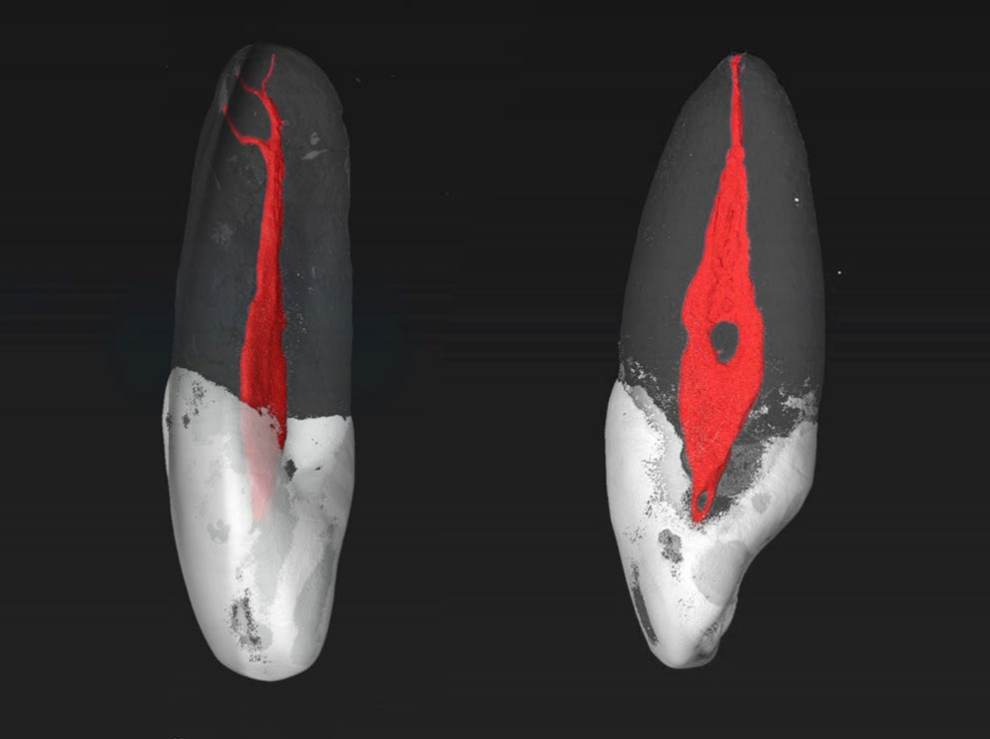
Micro-CT images of two maxillary lateral incisors with a RCC 1-1-1/2 with two accessory foramina (left) and a RCC of 1-2-1/1 with one accessory foramen (right). The enamel is colored with white, the pulp and root canal system with red and the dentin with a transparent gray

**Fig. 2:**
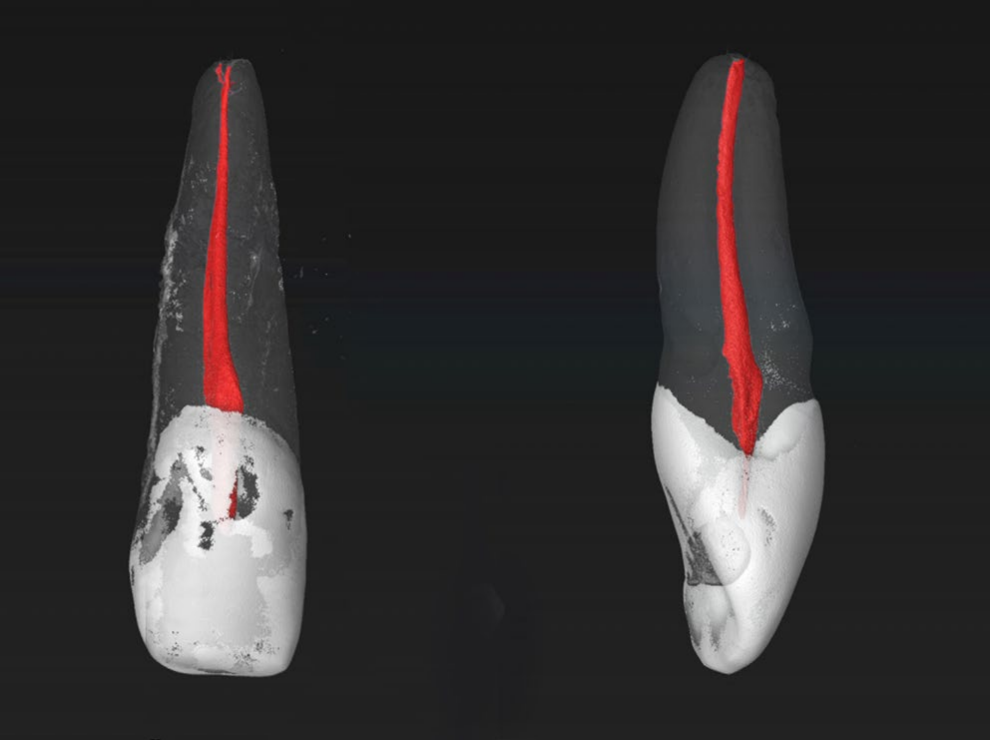
Micro-CT images of maxillary lateral incisors with a RCC of 1-1-1/1 and four accessory foramina (left) and no accessory foramina (right)

Most commonly, the MxLI presented one main foramen (diameter > 0.1 mm) (89.1%), two main foramina occurred less frequently (9.1%) and three were present in two teeth only (1.8%). A maximum of three accessory and connecting canals occurred per tooth. The accessory canals were found mostly in the apical third of the roots. In 72.7% of the MxLI examined, no accessory canals were present. In 20.0% of the samples, these canals showed up in the apical third of the root, less frequently in the middle third (6.4%), and only one tooth presented an accessory canal in the coronal third (0.9%). Examples of the samples are shown in Figs. 1 and 2.

## Discussion

The aim of this study was to investigate the internal morphology and root canal configuration of maxillary lateral incisors using micro-computed tomography. A wide variety of ex vivo and in vivo methods have been used to investigate the anatomical structures in teeth. Examination methods such as clearing and staining with dye or ink [1, 6–8, 20], magnification [6], and radiographic imaging [5] are mostly described in earlier studies. X-rays have a relatively low resolution compared to micro-CT and are difficult to reproduce, the intra- and interobserver reliability of the X-rays can vary considerably. Very fine, narrow structures such as accessory canals and foramina are often hardly visible with X-ray methodology. Clearing, staining, or cutting samples is a time-consuming process and may not continuously present the structures due to the thickness of the layer, cutting may involve artifacts, samples must be destroyed for examination and the area to be cut out cannot be examined. CBCT examinations seem to be a suitable method to visualize root canal morphology in vivo, as has already been shown in numerous studies [9, 11, 12]. The resolution of CBCT images is less accurate compared to micro-CT scans that show superior results in more detailed structures [27], so that accessory canals can be represented and tracked from their origin to their end. Modern three-dimensional ex vivo examinations such as Micro-CT allow for more detailed information and the representation of finer anatomical structures compared to in vivo examination methods such as cone beam computer tomography (CBCT) [27]. Micro-CT can therefore be called the gold standard for endodontic-morphological ex vivo research [15].

To the best of our knowledge, no study could be identified in the literature examining maxillary lateral incisors using micro-CT imaging for root canal configuration of a Swiss-German population, whereby the information obtained with micro-CT together with software imaging allows an easier evaluation compared to CBCT [15]. Micro-CT is a non-invasive and reproducible technique that enables both qualitative and quantitative analysis. So far, it is only possible to conduct ex vivo research on teeth and is sensible due to the high radiation exposure. Different classification systems have been introduced in the literature to describe the internal morphology of the roots and the root canal configuration. The most common RCC classifications have been developed by Weine et al. [17] differentiated into three different types of RCC, and Vertucci [1] classifying eight different types of RCC. Since modern investigation methods such as CBCT or micro-CT can represent precise structures, a need has arisen for a classification system that can capture or simplify these details. The RCC system of Briseño Marroquín et al. [18] defines the number of root canals in the thirds (coronal, middle, apical) of the root and the number of main foramina using the fourth digit. The present ex vivo study showed the most common RCC with 80.0% 1-1-1/1 (Ve I). This is in the low range but in line with previous reports with 78.05–100% [1, 5–13, 21–26, 28], with most authors reporting 1-1-1/1 RCC (Ve I) frequencies between 91 and 100% [1, 5, 7–12]. In addition, RCC were 1-2-1/1 (Ve III, 0.1-14.63%) [6–9, 28, 21, 26, 13], 1-1-2/2 (Ve V, 0.5–4.88%), and 2-2-1/1 (Ve II, 0.2–3.5%) with lower frequency in the reviewed literature [6–9, 28, 21, 13]. The reasons for the discrepancies in the different results could be the random selection of teeth, the ethnic origin as well as the examination method used. Only one group investigated accessory canals and apical foramina, while lateral canals were predominantly located (12.2%) in the apical third [6]. This is in line with the results of the present study with the most frequent occurrence of accessory canals (20.0%) in the apical third. Although the authors of the present study consider age and sex differences in the study of root canal configuration to be marginal and therefore irrelevant, the findings cannot neither be dismissed nor confirmed. The differences between the studies could generally be explained by the sample size, design, methodology, ethnicity of the sample, as well as differences in age and sex. While numerous factors such as wear, caries, occlusal trauma, and the time of eruption can influence root canal anatomy, age has also been found to be a decisive factor in morphologic changes regarding the volume of the root canal system, even by micro-CT [11, 12, 29]. However, the most important cause in the different studies could be the classification methodology used to investigate the internal root morphology and the root canal configuration. For the clinician, it is important to consider the occurrence of complex morphological conditions regarding the root canal configuration as well as the presence of accessory canals, which may not be mechanically accessible and reprocessed during the preparation of the root canal of maxillary lateral incisors. In this case, chemical root canal irrigation and adequate obturation of the root canal system are of particular importance, which, in combination with prior mechanical root canal preparation, have a significant influence on the success of non-surgical or surgical endodontic treatment.

## Limitations and strengths

Various limitations of the study should be mentioned. Although micro-CT examinations offer superior imaging details, they are still unsuitable for in vivo examinations due to the exclusive ex vivo analysis option and the high radiation exposure. This means that the transfer to clinical questions or patient cases is significantly limited. The study focused on a Swiss-German population, which limits the generalizability of the results to other populations with different genetic and environmental backgrounds. Also, the methodological application of the classification systems such as the three used in this study [1, 17, 18], although providing a lot of detailed information, may not cover all possible anatomical variations, especially regarding the description of specific anatomical structures such as isthmuses. Also, information on age and sex was unfortunately not available in this study, which could have an additional influence on the anatomical variation due to genetic or age-related remodeling processes. Nevertheless, this non-destructive and reproducible micro-CT technique, which allows both quantitative and qualitative analyses, makes an important contribution to the understanding of the complex internal morphology of maxillary lateral incisors, providing valuable insights to improve endodontic treatment planning.

## Conclusions

Within the limitations of the current ex vivo study, this micro-computed tomography analysis of maxillary lateral incisors provides clinicians with detailed insights and a better understanding of the challenges of root canal treatments. The following conclusions can be derived:

- The most frequently observed RCC in single-rooted maxillary lateral incisors of the Swiss-German population are 1-1-1/1 (Ve I, 80.0%), followed by 1-1-2/2 (Ve V, 7.3%), 1-2-1/1 (Ve III, 6.4%), 2-1-1/1 (Ve II, 1.8%) and 1-1-1/2 (1.8%).
- One physiological foramen is present in 89.1%, two in 9.1%, and three in 1.8%.
- A total of 27.3% of the samples showed accessory canals, mostly in the apical region (20.0%).

More than a quarter of the teeth examined showed RCC and the presence of accessory canals, which can significantly affect the approach to cleaning, shaping, and filling the root canal system.

## Data Availability

Data is contained within the article.
